# Supplementary material for: Complexome profiling of the Chlamydomonas psb28 mutant reveals TEF5 as an early PSII assembly factor
Source: Plant Cell. 2025 Mar 17;37(6):koaf055. doi: 10.1093/plcell/koaf055 (PMC12164590; doi:10.1093/plcell/koaf055)
Supplement: koaf055_Supplementary_Data [file koaf055_supplementary_data.zip › psb28_interactive.zip!/plots/intensity/qualityPlots/peptides/_Cre17.g702950.t1.2.html]

### Cre17.g702950.t1.2

Trivial name:   
SeqCoverage [median]: 21.10  
Mass: 24.43 kDa  
  
 

| Sequence | MissedCleavage | Mass [Da] | wtIntensity | muIntensity | Score |
| --- | --- | --- | --- | --- | --- |
| AHAHHTMDVESLSSSEGK | 0 | 1921.86 | 7512.67 | 36605.16 | 106.82 |
| AKAQLAMMTAPVVK | 1 | 1457.81 | 0.00 | 430.36 | 74.46 |
| AKAQLAMMTAPVVKDEWVR | 2 | 2143.13 | 0.00 | 800.76 | 88.26 |
| AQLAMMTAPVVK | 0 | 1258.68 | 9068.30 | 14107.74 | 200.09 |
| AQLAMMTAPVVKDEWVR | 1 | 1944.00 | 2353.67 | 17250.67 | 132.64 |
|  |  |  | 18934.6 | 69194.7 |  |
